# Correlation between endothelial dysfunction and occurrence of no-reflow in patients undergoing post-thrombolysis early invasive percutaneous intervention for ST-elevation myocardial infarction

**DOI:** 10.1186/s43044-022-00309-2

**Published:** 2022-09-30

**Authors:** Mohamed Abdel Wahab Elbendary, Mohamed Ayman Saleh, Sameh Saleh Sabet, Islam Bastawy

**Affiliations:** 1 Cardiology Department, Dar Elsalam Hospital, Cairo, Egypt; 2 Cardiology Department, Ain Shams University, Abbassia, Cairo, 11566 Egypt

**Keywords:** Coronary intervention, Endothelial dysfunction, Fibrinolysis, Flow-mediated dilatation, Myocardial blush grade, Myocardial infarction, No-reflow, TIMI flow

## Abstract

**Background:**

Endothelial dysfunction and no-reflow share microcirculatory obstruction as a common pathophysiological mechanism. This study evaluated the relationship between systemic peripheral endothelial dysfunction assessed by flow-mediated dilatation (FMD) of the brachial artery and no-reflow in patients with ST-segment elevation myocardial infarction (STEMI) who received successful fibrinolysis.

**Results:**

This study included 150 patients managed by the percutaneous coronary intervention (PCI) after successful fibrinolysis. Patients were divided according to coronary angiographic success into normal flow versus no-reflow groups. According to FMD measured through brachial artery ultrasound, patients were divided based on their endothelial function into endothelial dysfunction versus normal endothelial function. No-reflow occurred in 44 patients (29.3%). No-reflow patients had longer pain to door time (6.52 ± 1.82 vs 5.19 ± 1.85 h), more Killip class II (36.4% vs 16%, *p* = 0.006), and lower FMD (7.26 ± 1.92 vs 8.23 ± 2.76%, *p* = 0.036). Also, they showed more endothelial dysfunction; however, this difference was statistically nonsignificant (97.7% vs 87.7%, *p* = 0.055). One hundred and thirty-six patients (90.7%) had endothelial dysfunction. They were older (57.51 ± 5.92 vs 50.86 ± 4.55 years, *p* value ≤ 0.001), more smokers (41.2% vs 14.3%, *p* = 0.04). Patients with normal endothelial function had a more myocardial blush grade (MBG) 3 (78.6% vs 26.5%, *p* value = 0.001) in comparison with more MBG 2 in those with endothelial dysfunction (41.9% vs 14.3%, *p* value = 0.001). Endothelial dysfunction patients had nonsignificant more no-reflow (31.6% vs 7.1%, *p*-value: 0.06). There was a significant weak positive correlation between thrombolysis in myocardial infarction (TIMI) flow and FMD (*r* = 0.174, *p* = 0.033) and a significant moderate positive correlation between MBG and FMD (*r* = 0.366, *p* < 0.001). Patients with TIMI I flow had significantly lower FMD compared with patients with TIMI II and TIMI III flow post-PCI. FMD ≤ 6% could predict post-procedural TIMI I flow.

**Conclusions:**

In STEMI patients who underwent PCI within 24 h after successful fibrinolysis, those who had no-reflow showed worse peripheral systemic endothelial function as they had lower brachial artery FMD. Also, FMD showed a significant positive correlation with the post-procedural angiographic flow (TIMI flow and MBG). FMD ≤ 6% could predict TIMI I flow.

## Background

Normal endothelium regulates vascular motor tone through the release of nitric oxide. Also, it regulates vascular hemostasis and maintains blood flow by preventing platelet adhesion, leukocyte activation, and uncontrolled coagulation. On the other hand, dysfunctional endothelium plays a pivotal role in the development and progression of atherosclerosis. Also, it increases the vulnerability of atherosclerotic plaques and the risk of myocardial infarction [1–4]. Ruptured vulnerable atherosclerotic plaques show increased release and activity of vasoactive substances such as endothelin-1 [5]. Endothelin-1 could play a role in the pathogenesis of coronary no-reflow complicating coronary intervention through the mediation of functional microcirculatory obstruction [6].

No-reflow is related to increased cardiovascular morbidity and mortality. The pathophysiological mechanism of no-reflow is complex and multifactorial [7]. It includes mechanical microcirculatory obstruction due to distal thrombotic embolization and functional microcirculatory obstruction due to the release of vasoconstrictor mediators [6]. The link between endothelial dysfunction and no-reflow is still unclear. Theoretically, increased endothelin-1 may link endothelial dysfunction to no-reflow; however, clinical studies could not establish this relationship in ST-segment elevation myocardial infarction (STEMI) patients managed by the primary percutaneous intervention (PCI) [8].

This study evaluated the relationship between systemic peripheral endothelial dysfunction assessed by flow-mediated dilatation (FMD) of the brachial artery and no-reflow in patients with STEMI who received successful fibrinolysis.

## Methods

This prospective observational study was carried out in our institution in the period between September 2019 until August 2021. It included patients presented with STEMI within 12 h from chest pain who received successful fibrinolysis as the reperfusion modality when primary PCI is not available within 120 min; they were scheduled for invasive coronary angiography within 24 h from fibrinolysis according to European society of cardiology (ESC) guidelines [9]. Successful fibrinolysis was defined as (ST-segment resolution > 50% at 60–90 min; typical reperfusion arrhythmia; and disappearance of chest pain) [10]. The study excluded patients who refused to participate, patients with Killip class ≥ III, and patients referred for cardiothoracic surgical consultation after diagnostic angiography. The research ethics committee at Ain Shams University approved the study design (FMASU MD 352/2019), and all participants signed informed written consent. All patients received optimal medical therapy for STEMI as per ESC 2017 guidelines, unless any medication was contraindicated including loading anti-platelets (acetyl salicylic acid 300 mg and clopidogrel 300 mg) [9]. All participants were subjected to history taking to determine risk factors of coronary artery disease, established cardiovascular disease, and pain to door time in hours. Physical examination evaluated blood pressure and Killip class (where Killip class I showed no clinical signs of heart failure, Killip class II showed rales in the lungs, third heart sound (S3), and elevated jugular venous pressure, Killip class III showed acute pulmonary edema and Killip class IV had cardiogenic shock) [11]. Twelve-lead surface electrocardiogram (ECG) was done on admission to localize the territory of infarction (Anterior vs non-anterior) with the observation of baseline ST-segment elevation from J-Point. Our study included patients with successful fibrinolysis, so ECG was repeated 60–90 min after thrombolytic therapy to identify successful fibrinolysis (more than 50% ST segment resolution) [9, 10]. Transthoracic echocardiography excluded mechanical complications and evaluated left ventricular (LV) systolic function by the modified Simpson method. Blood samples were withdrawn on admission to measure creatine-kinase total (CK-total) and myocardial band (CK-MB).

### Coronary intervention

Coronary angiography was done through femoral or radial access using a 6F arterial sheath under local anesthesia, and intravenous pre-medications were given as needed. Infarct-related artery (IRA) was identified (left anterior descending artery (LAD), left circumflex artery (LCX), or right coronary artery (RCA)), then thrombolysis in myocardial infarction (TIMI) thrombus grade [12] and pre-procedural TIMI flow were recorded [13]. The choice of guiding catheter was according to coronary anatomy and operator choice. Percutaneous transluminal coronary angioplasty (PTCA) wire crossing of the lesion was done after giving parenteral anticoagulation using 100 IU/kg of unfractionated heparin. Balloon dilatation was done if needed, and coronary stenting was done using drug-eluting stents (DES). No-reflow was defined as (TIMI flow < III or myocardial blush grade (MBG) ≤ 1 despite the mechanical reopening of the IRA) [14]. TIMI flow grading system was defined as TIMI 0: complete occlusion of the infarct-related artery, TIMI I: some penetration of the contrast material beyond the point of obstruction but without perfusion of the distal coronary bed, TIMI II: perfusion of the entire infarct vessel into the distal bed but with delayed flow compared with a normal artery, TIMI III: full perfusion of the infarct vessel with the normal flow [13]. Blush grades are defined as follows: 0, absence of blush or contrast density; 1, minimal contrast density; 2, moderate contrast density, but less than that obtained with angiography of an artery unrelated to the infarct; and 3, normal contrast density comparable to that obtained with angiography of an artery unrelated to the infarct [15]. According to the reperfusion success, they were classified into two groups, no-reflow versus normal flow.

### Assessment of endothelial function

Assessment of endothelial function was done by measuring FMD before coronary angiography. Brachial artery duplex was performed using a General Electric (GE) vivid S5 machine using 8L-RS linear array transducer (4–13 MHz) with clear anterior and posterior intimal surfaces. Patients were lying in a supine position with their hands at the level of the heart, blood pressure cuff was placed over the antecubital fossa. Then the ultrasound probe was placed at a level just above the antecubital fossa and a baseline resting trans-sectional image of the brachial artery was obtained to measure its baseline diameter. After that, a blood pressure cuff was inflated to 40 mm Hg above systolic pressure for a standardized time (5 min), then the cuff was rapidly deflated to allow reactive hyperemia. Brachial artery diameter was recorded during the first minute after the pressure release to detect the maximal reactive hyperemia at the same point of the baseline diameter measurement. FMD is the percent change in diameter from baseline and was calculated by dividing the difference between hyperemic diameter and baseline diameter by the baseline diameter and then multiplying by 100. FMD% = (D2 − D1)/D1 × 100, Where D1 is baseline brachial artery diameter and D2 is hyperemic brachial artery diameter, and FMD% less than 10% was used to diagnose endothelial dysfunction [16]. The interventional cardiologists were blinded to brachial artery FMD% results. According to the presence of endothelial dysfunction, patients were classified into another two groups, endothelial dysfunction versus normal endothelial function.

### Statistical analysis

Data were collected, coded, and entered into IBM Statistical Package for Social Science (SPSS) version 23. Parametric numerical data were described by mean and standard deviation, while nonparametric numerical data was described by median and interquartile range (IQR). Non-numerical variables were presented as frequency and percentage. Analyses of qualitative variables were performed by chi-square. Parametric variables were analyzed by independent *t*-test, and nonparametric variables were analyzed by Mann–Whitney *U* test. Using receiver operating characteristic curves (ROC), we determined the prediction utility of brachial artery FMD% for no-reflow. The margin of error accepted was set to 5% by setting the confidence interval to 95%, so the *p*-value was considered significant if *p* < 0.05.

## Results

**Fig. 1:**
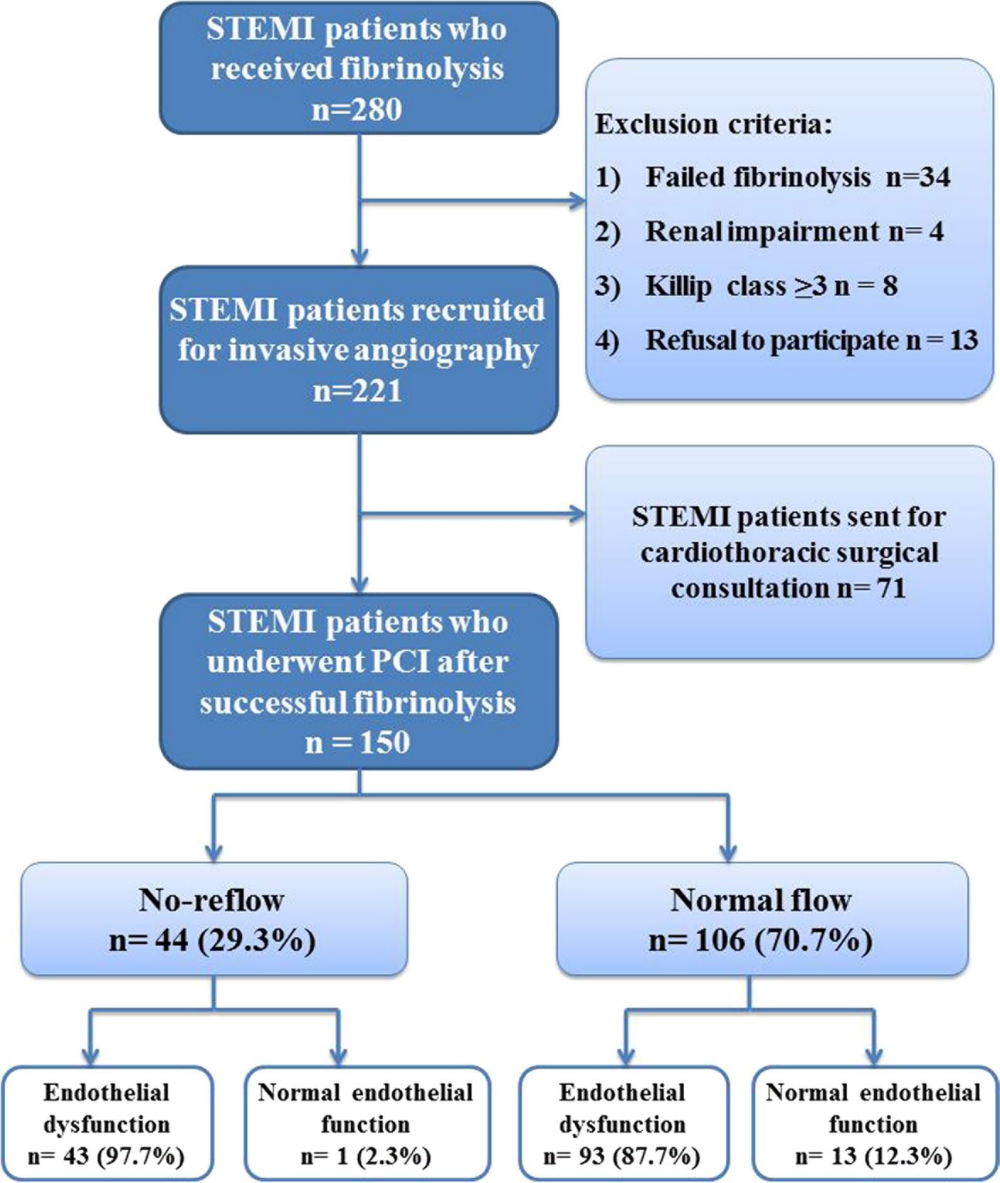
Flowchart of the study

The study flowchart is represented in Fig. 1. In the current study, one-hundred and fifty patients were managed by PCI after successful fibrinolysis and divided according to the success of reperfusion into two groups (Normal flow and no-reflow) with Table 1 showing the comparison between the two groups. No-reflow occurred in 44 patients (29.3%). There was no significant difference in comparison age, gender, risk factors for coronary artery disease, and previous cardiovascular disease. No-reflow group had significantly more anterior myocardial infarction, while those with normal flow had more non-anterior infarction. No-reflow patients showed significantly longer pain to door time (6.52 ± 1.82 vs 5.19 ± 1.85 h), more incidence of Killip class II (36.4% vs 16%, *p* = 0.006), higher CK-MB (136 (78.5–230.5) vs 69.5 (54–96) IU, *p* < 0.001), and lower ejection fraction (EF) (46.57 ± 9.93 vs 53.49 ± 8.99%, *p* < 0.001). On comparing the findings of brachial artery ultrasound, the no-reflow group had significantly lower FMD (7.26 ± 1.92 vs 8.23 ± 2.76%, *p* = 0.036). Also, they showed more endothelial dysfunction; however, this difference was statistically nonsignificant (97.7% vs 87.7%, *p* = 0.055).

**Table 1:** Comparison between normal flow and no-reflow groups

Variable	Normal flow *n* = 106	No-reflow *n* = 44	*p*-value
Baseline characteristics			
Age (years)	56.30 ± 6.07	58.32 ± 6.03	0.06•
Gender (%)			0.73*
Males	45 (42.5%)	20 (45.5%)	
Smoking (%)	42 (39.6%)	16 (36.4%)	0.7*
Hypertension (%)	44 (41.5%)	23 (52.3%)	0.22*
Diabetes mellitus (%)	28 (26.4%)	15 (34.1%)	0.34*
Family history (%)	23 (21.7%)	10 (22.7%)	0.89*
Previous PCI (%)	11 (10.4%)	6 (13.6%)	0.56*
Previous CABG (%)	1 (0.9%)	0 (0.0%)	0.51*
Previous myocardial infarction (%)	5 (4.7%)	4 (9.1%)	0.30*
Previous angina (%)	33 (31.1%)	17 (38.6%)	0.37*
Myocardial infarction presentation			
Territory of infarction (%)			0.01*
Anterior	49 (46.2%)	30 (68.2%)	
Non-anterior	57 (53.8%)	14 (31.8%)	
Pain to door (hours)	5.19 ± 1.85	6.52 ± 1.82	< 0.001•
SBP (mmHg)	118.92 ± 14.69	116.25 ± 15.93	0.32•
DBP (mmHg)	74.95 ± 11.92	70.23 ± 12.29	0.03•
Killip class (%)			0.006*
I	89 (84.0%)	28 (63.6%)	
II	17 (16.0%)	16 (36.4%)	
CK total (IU) Median (IQR)	563.5 (412 – 827)	974 (725.5 – 1685)	< 0.001 ≠
CK-MB (IU) Median (IQR)	69.5 (54 – 96)	136 (78.5 – 230.5)	< 0.001 ≠
EF (%)	53.49 ± 8.99	46.57 ± 9.93	< 0.001•
Angiographic findings			
Culprit vessel (%)			0.06*
LAD	58 (54.7%)	33 (75%)	
LCX	15 (14.1%)	4 (9.1%)	
RCA	33 (31.2%)	7 (15.9%)	
Balloon pre-dilatation	31 (29.2%)	13 (29.5%)	0.971*
Balloon post-dilatation	10 (9.4%)	4 (9.1%)	0.95*
Thrombus burden grade			< 0.001*
I	79 (74.5%)	16 (36.4%)	
II	27 (25.5%)	24 (54.5%)	
III	0 (0.0%)	4 (9.1%)	
Baseline TIMI flow			< 0.001*
I	3 (2.8%)	13 (29.5%)	
II	47 (44.3%)	24 (54.5%)	
III	56 (52.8%)	7 (15.9%)	
Ultrasound findings			
Baseline brachial artery diameter (cm)	0.44 ± 0.04	0.45 ± 0.05	< 0.001•
Brachial artery diameter after release (cm)	0.48 ± 0.05	0.48 ± 0.06	0.97•
Endothelial dysfunction (%)	93 (87.7%)	43 (97.7%)	0.06•
FMD (%)	8.23 ± 2.76	7.26 ± 1.92	0.03•

*CABG* Coronary artery bypass graft, *CK* Creatine kinase, *CK-MB* Creatine kinase myocardial band, *DBP* Diastolic blood pressure, *EF* Ejection fraction, *FMD* Flow-mediated dilatation, *IQR* Interquartile range, *IU* International unit, *LAD* Left anterior descending artery, *LCX* Left circumflex artery, *PCI* Percutaneous intervention, *RCA* Right coronary artery, *SBP* Systolic blood pressure

*: Chi-square test; •: Independent *t*-test ≠: Mann–Whitney test

Patients were divided according to FMD into one-hundred and thirty-six patients (90.7%) with endothelial dysfunction, while 14 patients (9.3%) had a normal endothelial function with Table 2 showing the comparison between the two groups. There was no significant difference in comparing gender, hypertension, diabetes mellitus, Killip class, pain to door time, and EF between both groups, while those with endothelial dysfunction were significantly older (57.51 ± 5.92 vs 50.86 ± 4.55 years, *p* value ≤ 0.001), more smokers (41.2% vs 14.3%, *p* = 0.04), and had a significantly higher incidence of family history of premature coronary artery disease (19.9% vs 42.9%, *p* = 0.04). On comparing angiographic findings between both groups, there was no significant difference in baseline coronary TIMI flow and thrombus burden before PCI or post-PCI TIMI flow. However, on comparing MBG between both groups, patients without endothelial dysfunction had a higher incidence of MBG 3 (78.6% vs 26.5%) in comparison with a higher incidence of MBG 2 in those with endothelial dysfunction (41.9% vs 14.3%). However, on comparing the success of myocardial perfusion, endothelial dysfunction patients showed more no-reflow; however, this was not statistically significant (31.6% vs 7.1%, *p*-value: 0.06).

**Table 2:** Comparison between endothelial dysfunction and normal endothelial function groups

Variable	Normal endothelial function *n* = 14	Endothelial dysfunction *n* = 136	*p*-value
*Baseline characteristics*			
Age (years)	50.86 ± 4.55	57.51 ± 5.92	< 0.001•
Gender (%)			0.09*
Males	5 (35.7%)	80 (58.8%)	
Smoking (%)	2 (14.3%)	56 (41.2%)	0.04*
Hypertension (%)	5 (35.7%)	62 (45.6%)	0.47*
Diabetes mellitus (%)	2 (14.3%)	41 (30.1%)	0.21*
Family history (%)	6 (42.9%)	27 (19.9%)	0.04*
Previous PCI (%)	1 (7.1%)	16 (11.8%)	0.6*
Previous CABG (%)	0 (0.0%)	1 (0.7%)	0.74*
Previous myocardial infarction (%)	0 (0.0%)	9 (6.6%)	0.32*
Previous angina (%)	3 (21.4%)	47 (34.6%)	0.32*
*Myocardial infarction presentation*			
Territory of infarction (%)			0.58*
Anterior	4 (28.6%)	75 (55.1%)	
Non-anterior	10 (71.5%)	61 (44.8%)	
Pain to door (hours)	5.64 ± 2.17	5.57 ± 1.92	0.89•
SBP (mmHg)	117.50 ± 13.97	118.20 ± 15.21	0.86•
DBP (mmHg)	73.57 ± 9.49	73.57 ± 12.46	0.99•
Killip class			0.46*
I	12 (85.7%)	105 (77.2%)	
II	2 (14.3%)	31 (22.8%)	
*Angiographic findings*			
Culprit vessel (%)			0.3*
LAD	6 (42.8%)	85 (62.5%)	
LCX	2(14.4%)	17(12.5%)	
RCA	6 (42.8%)	34 (25%)	
Thrombus burden grade			0.36*
I	7 (50.0%)	88 (64.7%)	
II	7 (50.0%)	44 (32.4%)	
III	0 (0.0%)	4 (2.9%)	
Baseline TIMI flow			0.558*
I	2 (14.3%)	14 (10.3%)	
II	8 (57.1%)	63 (46.3%)	
III	4 (28.6%)	59 (43.4%)	
Post-PCI TIMI flow			0.37*
I	0 (0.0%)	8 (5.9%)	
II	1 (7.1%)	23 (16.9%)	
III	13 (92.9%)	105 (77.2%)	
Success of epicardial reperfusion by TIMI flow			0.17*
Non TIMI III	1 (7.1%)	31 (22.8%)	
TIMI III	13 (92.9%)	105 (77.2%)	
Myocardial blush grade			0.001*
0	0 (0.0%)	21 (15.4%)	
1	1 (7.1%)	22 (16.2%)	
2	2 (14.3%)	57 (41.9%)	
3	11 (78.6%)	36 (26.5%)	
Success of myocardial reperfusion (by MBG)			0.06*
MBG 0–1	1 (7.1%)	43 (31.6%)	
MBG 2–3	13 (92.9%)	93 (68.4%)	

*CABG* Coronary artery bypass graft, *DBP* Diastolic blood pressure, *LAD* Left anterior descending artery, *LCX* Left circumflex artery, *MBG* Myocardial blush grade, *PCI* Percutaneous intervention, *RCA* Right coronary artery, *SBP* Systolic blood pressure, *TIMI* Thrombolysis in myocardial infarction

*: Chi-square test; •: Independent *t*-test

**Fig. 2:**
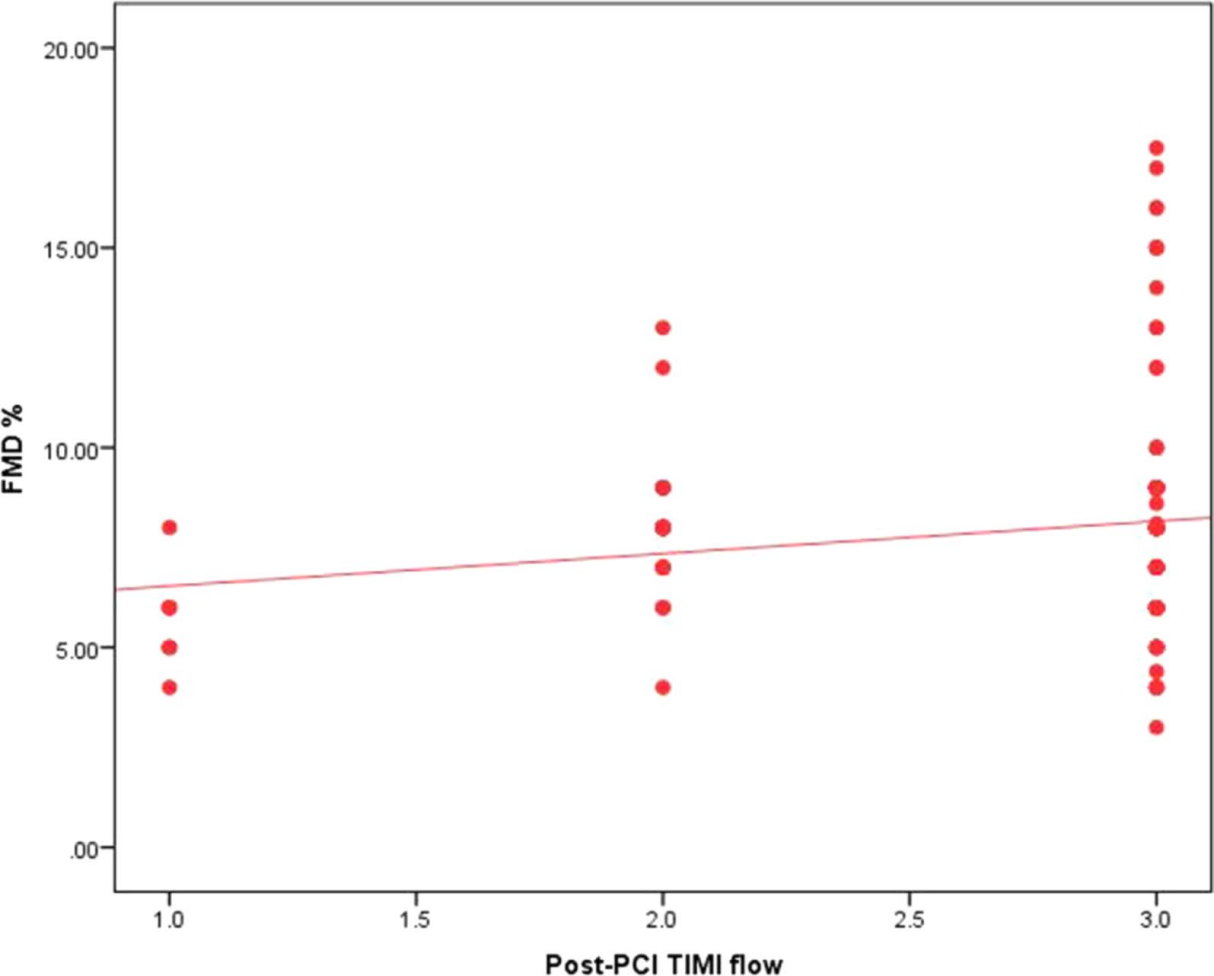
Correlation between FMD and TIMI flow

**Fig. 3:**
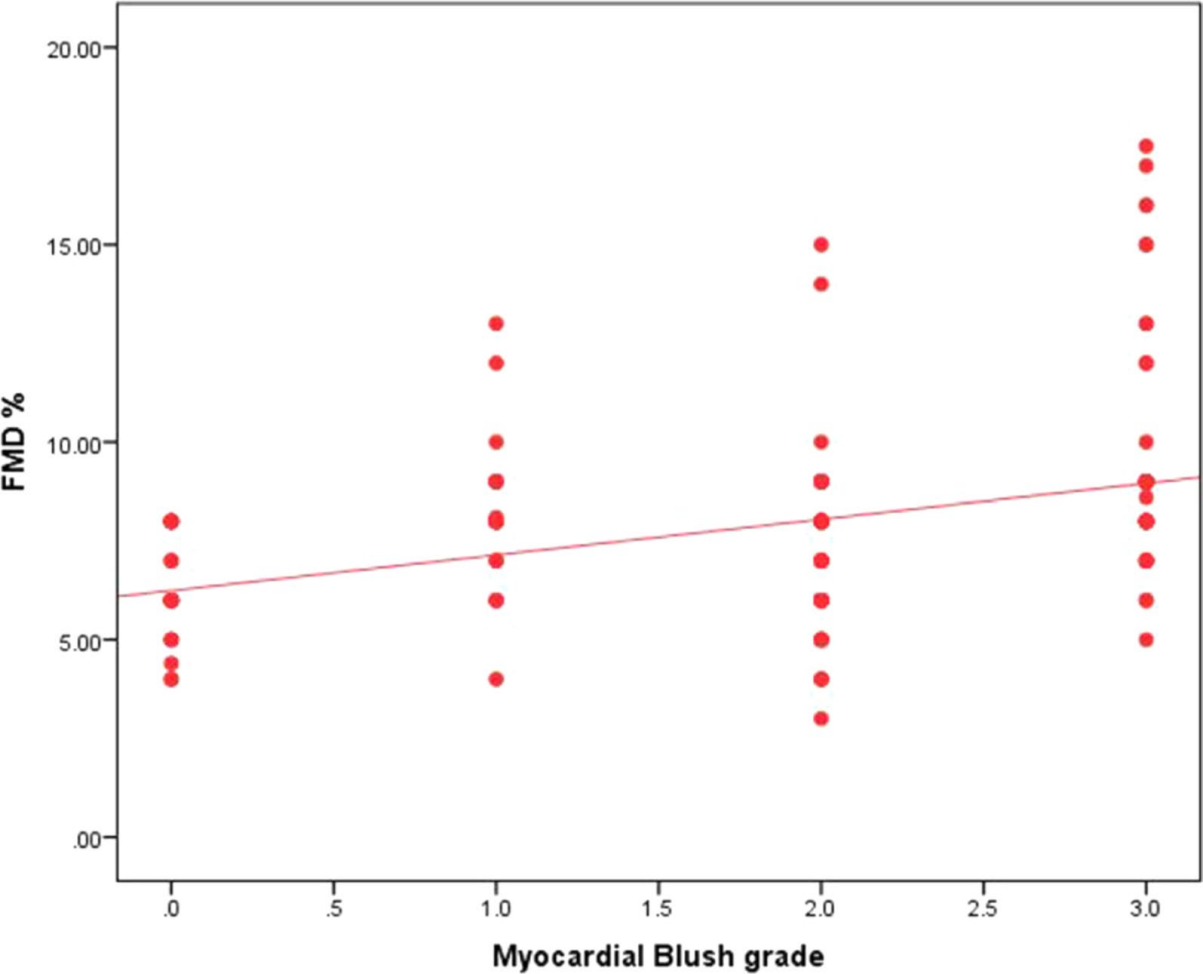
Correlation between FMD and MBG

There was no significant correlation between FMD and age (*r* = − 0.101, *p* = 0.217) or pain to door (*r* = 0.019, *p* = 0.821), while there was significant weak positive correlation between post-procedural TIMI flow and FMD (*r* = 0.174, *p* = 0.033) (Fig. 2) in addition to significant moderate positive correlation between MBG and FMD (*r* = 0.366, *p* < 0.001) (Fig. 3).

On comparing FMD percentage between TIMI flow grades, post hoc analysis showed that the patients with TIMI I flow post-PCI had significantly lower FMD compared with patients with TIMI II and TIMI III flow post-PCI, and there was no significant difference between FMD values between patients with TIMI II and TIMI III flow post-PCI as shown in Table 3. Furthermore, post hoc analysis comparing FMD percentage between MBG showed that patients who had MBG 3 had significantly higher FMD percentage compared to patients who ended up with MBG 2, MBG 1 and MBG 0 as shown in Table 4. ROC curve analysis showed that FMD ≤ 6% is the best cutoff value to predict post-procedural TIMI I flow as shown in Table 5.

**Table 3:** Comparing FMD% between post-procedural TIMI flow grades

FMD %	Post-PCI TIMI flow			*p*-value	P1 (I vs II)	P2 (I vs III)	P3 (II vs III)
	I	II	III				
	*n* = 8	*n* = 24	*n* = 118				
Mean ± SD	5.63 ± 1.19	7.96 ± 1.83	8.10 ± 2.7	0.03	0.025	0.008	0.805

*FMD%* Flow-mediated dilatation percentage, *PCI* Percutaneous intervention, *SD* Standard deviation, *TIMI* Thrombolysis in myocardial infarction

**Table 4:** Comparing FMD% between post-procedural MBG

FMD %	Grade 0	Grade 1	Grade 2	Grade 3	*p*-value
	*n* = 21	*n* = 23	*n* = 59	*n* = 47	
*Myocardial blush grade*					
Mean ± SD	6.26 ± 1.38	8.18 ± 1.90	7.24 ± 2.08	9.47 ± 3.01	< 0.001
Range	4–8	4–13	3–15	5–17.5	

Grade 0 vs. grade 1	Grade 0 vs grade 2	Grade 0 vs grade 3	Grade 1 vs grade 2	Grade 1 vs grade 3	Grade 2 vs grade 3
*Post hoc analysis*					
0.007	0.099	< 0.001	0.101	0.030	< 0.001

*FMD%* Flow-mediated dilatation percentage, *PCI* Percutaneous intervention, *SD* Standard deviation, *TIMI* Thrombolysis in myocardial infarction, *vs* versus

**Table 5:** FMD cutoff value to predict TIMI I flow post-PCI

Cut off point	AUC	Sensitivity (%)	Specificity (%)	+ PV	− PV
≤ 6	0.837	87.50	74.65	16.3	99.1

*AUC* Area under curve,  − *PV* Negative predictive value, + *PV* Positive predictive value

## Discussion

In the current study, patients who presented with STEMI within 12 h from chest pain onset and had PCI after successful fibrinolysis showed two key findings. The no-reflow group had a significantly lower brachial artery FMD measured before invasive angiography within 24 h from STEMI, and there was a significant positive correlation between brachial artery FMD percentage and post-procedural angiographic flow (TIMI flow and MBG). Also, FMD ≤ 6% could be able to predict TIMI I flow.

In the current study, the assessment of systemic endothelial function was done by measuring brachial arterial FMD which correlates with coronary endothelial function [17]. Endothelial dysfunction results in decreased production of nitric oxide with increased vascular tone and microvascular resistance due to increased activity of vasoactive substances such as endothelin-1. Coronary endothelial dysfunction shares in the pathophysiology of atherosclerosis and increases the vulnerability of atherosclerotic plaques increasing the risk of an acute coronary syndrome [1–5]. In return, myocardial infarction-induced endothelial damage aggravates endothelial dysfunction resulting in functional microvascular obstruction through increased microvascular resistance that increases the risk of no-reflow [2, 7]. Pathogenesis of no-reflow is multifactorial and several mechanisms are involved including high thrombus burden with distal thromboembolism, prolonged duration of ischemia, and reperfusion injury [7]. This may explain a worse post-procedural TIMI flow and MBG with declining brachial artery FMD. However, in comparing the presence of systemic endothelial dysfunction diagnosis between the no-reflow and normal flow groups, the difference was not statistically significant. Some factors could explain this finding. First, only a small percentage of myocardial infarction patients have normal endothelial dysfunction as in the current study (nearly only 10%). Second, despite the correlation between systemic and coronary endothelial function, local endothelial damage with myocardial infarction increases coronary endothelial dysfunction [2]. Third, the multifactorial pathogenesis of no-reflow includes endothelial dysfunction as a contributing factor among other variables. One of these factors is the prolonged ischemic time as in the current study; however, FMD did not correlate with ischemic time.

In comparison with the current study, Levi et al. [8] study did not show a difference in endothelial dysfunction between reflow and no-reflow patients as assessed by measuring peripheral arterial tonometry 2–3 days after primary PCI. However, this does not rule out this relationship as the number of patients in this study was small. On the other side, Rashed et al. [18] study concluded that FMD < 11% could predict no-reflow. Also, the study by Vasilieva et al. [19] showed that the incidence of spontaneous thrombolysis was more associated with better FMD. Moreover, the study by Bravo Baptista et al. [20] showed that endothelial dysfunction was associated with microvascular obstruction and larger infarction size.

The clinical importance of the findings of the current study may be summarized in two points. First, although endothelial dysfunction is not the only determinant of no-reflow, it is correlating with post-procedural angiographic success. So, measures taken to improve endothelial function may have a beneficial effect on angiographic success in the setting of acute myocardial infarction. Many studies evaluated the effect of different drugs on improving endothelial function [3, 21–23]. Among these drugs, statins and angiotensin-converting enzyme inhibitors have proven beneficial effects [24]. Second, as the current guidelines recommend invasive angiography within 2–24 h, there is enough time to measure brachial artery FMD that could predict TIMI I flow if ≤ 6 which may direct future research to study the preventive measures against no-reflow in this particular group.

### Study limitations

One of the limitations of the study is that STEMI patients mostly have endothelial dysfunction. In the current study, nearly 10% only showed normal endothelial dysfunction. This small number of patients could explain that despite more no-reflow in those with endothelial dysfunction, this finding was statistically non-significant. The limitations of endothelial dysfunction assessment using this technique include the need for standardized protocols by trained personnel to maintain reproducibility, accuracy, and reliability [25, 26].

## Conclusions

In STEMI patients who underwent PCI within 24 h after successful fibrinolysis, those who had no-reflow showed worse peripheral systemic endothelial function as they had lower brachial artery FMD. Also, FMD showed a significant positive correlation with the post-procedural angiographic flow (TIMI flow and MBG). FMD ≤ 6% could predict TIMI I flow on PCI to IRA.

These findings emphasize the relationship between endothelial dysfunction and no-reflow. Also, they may point to a potential reduction of no-reflow through improving endothelial dysfunction.

## Data Availability

The datasets used and analyzed during the current study are available from the corresponding author on reasonable request.

## Abbreviations

AUC: Area under curve
CABG: Coronary artery bypass graft
CK: Creatine kinase
CK-MB: Creatine kinase myocardial band
DBP: Diastolic blood pressure
DES: Drug-eluting stents
ECG: Electrocardiography
ESC: European society of cardiology
EF: Ejection fraction
FMD: Flow-mediated dilatation
GE: General electric
IQR: Interquartile range
IRA: Infarcted related artery
LAD: Left anterior descending artery
LCX: Left circumflex artery
LV: Left ventricle
MBG: Myocardial blush grade
PCI: Percutaneous intervention
PTCA: Percutaneous transluminal coronary angioplasty
RCA: Right coronary artery
ROC: Receiver operating characteristic curves
SBP: Systolic blood pressure
STEMI: ST-segment elevation myocardial infarction
SPSS: Statistical package for social science
TIMI: Thrombolysis in myocardial infarction
